# Human‐mediated evolution in a threatened species? Juvenile life‐history changes in Snake River salmon

**DOI:** 10.1111/eva.12468

**Published:** 2017-05-19

**Authors:** Robin S. Waples, Anna Elz, Billy D. Arnsberg, James R. Faulkner, Jeffrey J. Hard, Emma Timmins‐Schiffman, Linda K. Park

**Affiliations:** ^1^ Northwest Fisheries Science Center National Marine Fisheries Service National Oceanic and Atmospheric Administration Seattle WA USA; ^2^ Department of Fisheries Resources Management Nez Perce Tribe Lapwai ID USA; ^3^ Department of Genome Sciences University of Washington Seattle WA USA

**Keywords:** anthro‐evolutionary species, endangered species, heritability, juvenile growth rate, life‐history evolution, phenotypic plasticity

## Abstract

Evaluations of human impacts on Earth's ecosystems often ignore evolutionary changes in response to altered selective regimes. Freshwater habitats for Snake River fall Chinook salmon (SRFCS), a threatened species in the US, have been dramatically changed by hydropower development and other watershed modifications. Associated biological changes include a shift in juvenile life history: Historically essentially 100% of juveniles migrated to sea as subyearlings, but a substantial fraction have migrated as yearlings in recent years. In contemplating future management actions for this species should major Snake River dams ever be removed (as many have proposed), it will be important to understand whether evolution is at least partially responsible for this life‐history change. We hypothesized that if this trait is genetically based, parents who migrated to sea as subyearlings should produce faster‐growing offspring that would be more likely to reach a size threshold to migrate to sea in their first year. We tested this with phenotypic data for over 2,600 juvenile SRFCS that were genetically matched to parents of hatchery and natural origin. Three lines of evidence supported our hypothesis: (i) the animal model estimated substantial heritability for juvenile growth rate for three consecutive cohorts; (ii) linear modeling showed an association between juvenile life history of parents and offspring growth rate; and (iii) faster‐growing juveniles migrated at greater speeds, as expected if they were more likely to be heading to sea. Surprisingly, we also found that parents reared a full year in a hatchery produced the fastest growing offspring of all—apparently an example of cross‐generational plasticity associated with artificial propagation. We suggest that SRFCS is an example of a potentially large class of species that can be considered to be “anthro‐evolutionary”—signifying those whose evolutionary trajectories have been profoundly shaped by altered selective regimes in human‐dominated landscapes.

## 1 INTRODUCTION

As a consequence of major anthropogenic changes to all of the planet's ecosystems (e.g., Vitousek, Mooney, Lubchenco, & Melillo, 1997), it has been suggested that we are now facing a biodiversity extinction crisis to rival the most extreme in the planet's history (Barnosky et al., 2011). Populations and species not driven to extinction are forced to cope with greatly altered environmental conditions. Some organisms can shift their distributions toward locations with more favorable conditions (Pinsky, Worm, Fogarty, Sarmiento, & Levin 2013; Poloczanska et al., 2013); others must rely on phenotypic plasticity and/or evolution (Chevin, Lande, & Mace, 2010; Ernande, Dieckmann, & Heino, 2004; Reed, Schindler, & Waples, 2011). This predicament faced by much of Earth's biodiversity raises challenging questions regarding preservation versus conservation.

Preservation is generally concerned with saving specific types of organisms, while conservation focuses more on maintaining fundamental processes such as natural selection and adaptation. From one perspective it is important to try to minimize human influences; the other perspective might embrace human‐mediated changes to biodiversity, provided the changes allow organisms to better cope with their strongly altered environments. These issues are particularly germane to species covered by national protected‐species legislation, such as the U.S. Endangered Species Act (ESA), Canada's Species at Risk Act (SARA), or Australia's Endangered Species Protection Act (ESPA). Should greatly altered ecosystems be considered the “new natural,” and if so is it desirable when species adapt to them? But what happens if environmental degradation can eventually be reversed? In an ironic twist of fate, some species might find themselves at least temporarily maladapted to the restored, quasi‐pristine environments under which they originally evolved.

**Figure 1:**
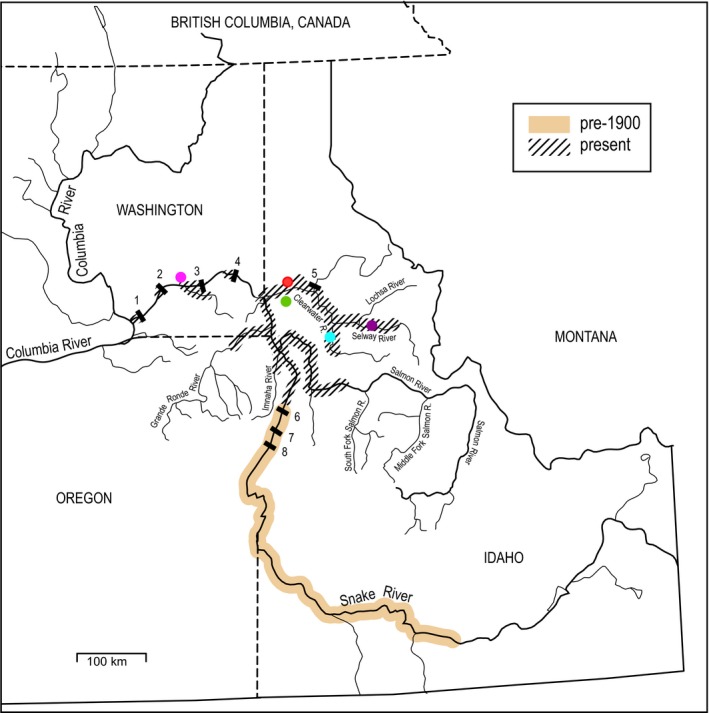
Map of the Snake River basin showing the major historic and current spawning areas for fall Chinook salmon. Colored circles are sampling sites. Adults were spawned in 2007–2009 at the Nez Perce Tribal Hatchery (NPTH, red); Lyons Ferry Hatchery (LFH, pink) is also shown. Juveniles were reared, sampled, and released at the NPTH (north and south ponds, red), Cedar Flats (purple), Luke's Gulch (blue), and North Lapwai Valley (east and west ponds, green). Numbers indicate major Snake River dams: 1, Ice Harbor; 2, Lower Monumental; 3, Little Goose; 4, Lower Granite; 5, Dworshak; 6, Hells Canyon; 7, Oxbow; 8, Brownlee. Salmon migrating to or from the Snake River also have to pass four large dams on the Columbia River (not shown)

Fall‐run Chinook salmon (*Oncorhynchus tshawytscha*) from the Snake River in the US (SRFCS; so named because adults on their spawning migration enter freshwater in the fall) are a poster‐child example of a population that has experienced manifold environmental changes imposed by humans. Historically, adults left the Pacific Ocean in late summer and then swam over 500 km up the Columbia River and almost 1,000 km farther up the Snake River to spawn in areas near the current location of Twin Falls, Idaho (Parkhurst, 1950). Since three dams without fish passage facilities were constructed in Hells Canyon (1959–1967), SRFCS have been constrained to <20% of their historical range (Figure 1).

Most of the remnant population has to traverse eight major hydroelectric dam/reservoir systems (four in the Snake and four in the Columbia), both as juveniles and adults, before they can complete their life cycle and spawn in the region above Lower Granite Dam (Connor, Burge, Waitt, & Bjornn, 2002). In addition to imposing mortality on both juveniles and adults (Kareiva, Marvier, & McClure, 2000), hydropower development alters river ecosystems in significant ways that select for individuals with different traits (e.g., stamina; migration timing; predator–prey relationships; Waples, Zabel, Scheuerell, & Sanderson, 2008). Reservoirs behind the dams support large populations of dozens of non‐native fish species, including important predators of juvenile salmon (Fritts & Pearsons, 2004; Sanderson, Barnas, & Rub, 2009). Exploitation of Columbia and Snake River salmon by Native Americans for subsistence and trade dates back at least 10,000 years (Chatters, Butler, Scott, Anderson, & Neitzel, 1995); however, harvest increased rapidly following European settlement, and for the better part of a century SRFCS have experienced annual harvest rates of 50%–80% or more (Connor et al., 2016; Ford et al., 2015). Hatchery propagation of SRFCS began in the 1980s, and since 2000 an average of about five million juvenile hatchery salmon have been released each year (W. Connor, U.S. Fish and Wildlife Service, Ahsahka, Idaho pers com).

Following alarming declines in abundance coincident with construction of four dams on the lower Snake River (1961–1975), as well as evidence that strays from a Columbia River hatchery program were entering the Snake River in large numbers, the U.S. National Marine Fisheries Service (NMFS) listed SRFCS as a threatened species under the ESA in 1992 (Waples, Jones, Beckman, & Swan, 1991; NMFS 1992). Recent assessments indicate that the status of SRFCS has improved markedly over the past 25 years (about 20,000–50,000 natural spawners in 2010–2015, many times the number at the time of ESA listing), but significant concerns remain about long‐term effects of a high proportion (~55%–75%) of artificially propagated individuals in the adult population (Ford et al., 2015).

Associated with the dramatic anthropogenic changes to its habitats, SRFCS have also undergone rapid change in juvenile life history. Biologists have long recognized two major Chinook salmon life histories based on the age at which they become smolts and migrate to sea: smolting in the first year of life (subyearling strategy), or smolting at age 1, after spending a full year in freshwater (yearling strategy) (Gilbert, 1912; Healey, 1991). Historical records (for periods prior to about 1970) failed to find any evidence for adult SRFCS that had adopted the yearling strategy (reviewed by Williams, Zabel, Waples, Hutchings, & Connor, 2008). This likely reflects three factors associated with historical SRFCS habitats. First, this part of the Snake River basin receives ground water at ~15 C from the Eastern Snake Plain Aquifer, which moderates river temperatures and promotes early emergence and rapid growth of juveniles (Chandler, Groves, & Bates, 2001). Second, by midsummer temperature in most of the mainstem Snake River probably exceeded the thermal tolerance of juvenile Chinook salmon (Connor et al., 2002, 2016; Waples et al., 1991). Therefore, juveniles that remained in the river and did not migrate rapidly to sea as subyearlings likely had poor survival. Finally, the historically free‐flowing Snake/Columbia River system provided rapid delivery to the estuary even for juveniles that had to migrate many hundreds of kilometers.

Incidence of the yearling life history has increased in recent decades, to the extent that up to 75% of returning adult females have been produced by yearling migrants (Williams et al., 2008). Environmental factors likely to have influenced this life‐history change include colder water and less favorable growing conditions in the remnant habitat below Hells Canyon Dam (Dauble, Hanrahan, Geist, & Parsley, 2003), as well as availability of large reservoirs behind the lower Snake River and Columbia River dams, which provide convenient holding and overwintering habitat for juveniles that did not exist historically. In particular, summer releases of cold water from upstream reservoirs provide a thermal buffer in Lower Granite Dam Reservoir, which is the first reservoir most wild SRFCS encounter on their downstream migration (Connor, Sneva, Tiffan, Steinhorst, & Ross, 2005).

Currently, wild SRFCS fry emerge in late April to early May in the middle Snake River mainstem and in June in the relatively cooler Clearwater River (Connor et al., 2002). Juveniles start a discontinuous downstream dispersal along the shorelines or continuous movement offshore in the free‐flowing river as they grow and start to take on morphological features characteristic of smoltification (Connor, Steinhorst, & Burge, 2003). Connor et al. (2002) reported that arrival of subyearling fall Chinook salmon at Lower Granite Dam peaked in July. Once in the reservoirs of the lower Snake River, most subyearlings continue migration directly to sea, but some slow or temporarily stop migration to seek further growth opportunities (Connor et al., 2003). Many of the fish that temporarily delay migration will continue in time to reach the Columbia River estuary by late fall. The majority of fish that do not migrate to sea by late fall will overwinter in the reservoirs of the lower Snake River and complete their migration as yearlings in the following spring, thus taking on a yearling or “reservoir‐type” life history (Connor et al., 2002, 2005; Tiffan, Kock, Connor, Mullins, & Steinhorst, 2012). Reservoir‐type fish undergo partial smoltification during the winter holdover period and complete the process in the spring (Connor et al., 2005; Tiffan et al., 2012).

**Figure 2:**
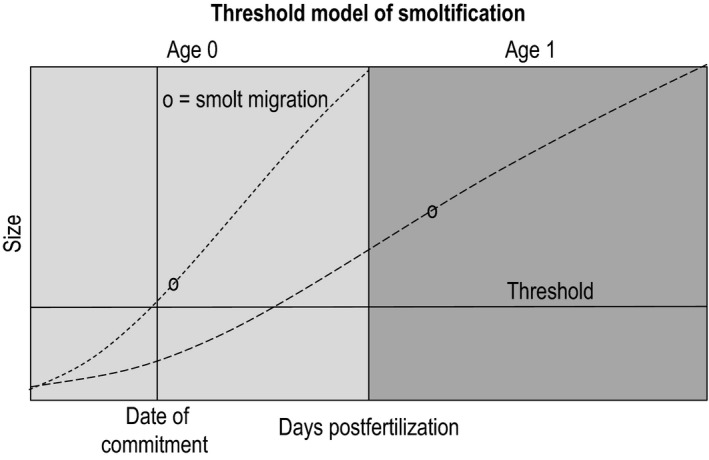
A schematic diagram of a threshold model for expression of a life‐history trait (in this case, timing of juvenile migration to the sea = smoltification). By a certain date (vertical line) in its first year in freshwater (age 0), a juvenile must commit to either undergo the process of smoltification and seaward migration, or remain in freshwater for another year. Whether an individual expresses the trait is determined by whether its phenotype (which could be size, physiological status, or growth rate during a critical period) meets a threshold (horizontal line). As applied to Snake River fall Chinook salmon, this model determines whether the individual migrates at age 0 (those that meet the initial threshold) or remains another year in freshwater (those that do not). Those that do not meet the initial threshold and survive to age 1 are larger when they migrate to sea and generally have higher marine survival and mature at earlier ages than age 0 smolts. Thus, factors that affect whether individuals meet the threshold to smolt at age 0 also influence the selective regimes experienced by those individuals throughout the rest of their life cycle

The threshold trait model of quantitative genetics provides a useful framework for considering the type of behavioral dichotomy associated with smolt age. As applied to SRFCS, this model postulates that an individual fish must reach a critical threshold related to size or physiological condition before migrating to sea; alternatively, the threshold could be growth rate during a critical seasonal period (Beckman, Larsen, & Dickhoff, 2003). Individuals that grow fast reach the threshold in their first year, while those that do not spend another year in freshwater (Figure 2).

Available evidence suggests that both environmental and genetic factors are important for SRFCS. Water temperature and flow can affect both growth rate and migration timing in juvenile Chinook salmon (Connor & Burge, 2003; Sykes, Johnson, & Shrimpton, 2009; Taylor, 1990), and Perkins and Jager (2011) found that a threshold model that included migration age as a function of growth rate and cues for temperature and photoperiod explained the majority of variation in empirical estimates of the percentage of yearling smolts in SRFCS reported by Connor et al. (2002). On the other hand, juvenile migration timing, like many other life‐history traits, is heritable in Chinook salmon (Carlson & Seamons, 2008; Clarke, Withler, & Shelbourn, 1994), and rapid evolution of juvenile life history has been reported in this species (Quinn, Unwin, & Kinnison, 2000).

Williams et al. (2008) concluded that the yearling smolt strategy in SRFCS is now selectively favored, presumably because of the substantial survival advantage conferred by their much larger size (Figure 2). SRFCS juveniles migrating downstream in the Snake River after overwintering in a reservoir averaged 222 mm fork length compared to 112 and 139 mm for age 0 migrants of hatchery and wild origin, respectively (Connor et al., 2005). The yearling smolts also migrate much earlier in the season, peaking in March and April (Connor et al., 2005) rather than midsummer for the subyearlings. These substantial changes in size and timing of migration create dramatically different selective regimes, which in turn mean that an evolutionary response by the population can be expected, although to date no empirical evidence for such evolution exists. Thus, while it is clear that environmental conditions have affected growth rate and hence juvenile life history in SRFCS, it is not known to what degree these plastic responses have been accompanied by genetic change.

This is an important issue to resolve because it directly affects conservation and management of this federally protected species. Mainstem dams on the Columbia/Snake system are large structures, but with functional lifetimes of a century or so they are ephemeral on geologic/evolutionary timescales. Notably, in 2014 Wanapum Dam, a large Columbia River dam built in 1959, developed a large crack that required lowering water levels as a precautionary measure (http://www.kulr8.com/story/25930717/wanapum-dam-constructions-starts-fixing-crack). Environmental groups, various state agencies, and Northwest tribes have called for removal or breaching of four large dams on the lower Snake River (USACE 2010). This would restore several hundred kilometers of free‐flowing river and more closely approximate historical habitat conditions. If recent changes in juvenile life history only reflect plastic responses to altered environments, SRFCS should be able to quickly adjust to the changes. However, if the population has genetically adapted its life history and enhanced the fraction of selectively favored yearling migrants, then its fitness could drop suddenly when more natural habitat conditions are restored, especially if the restored free‐flowing river no longer provides suitable overwintering habitat for individuals that do not migrate as subyearlings.

Williams et al. (2008) posed this dilemma but could not resolve it with available data. In this study, we use a three‐pronged approach to empirically evaluate evidence for genetic change in juvenile life history of SRFCS. First, we postulate that if smolt age is under at least partial genetic control, parents who were subyearling migrants should produce offspring that grow faster and hence are more likely to meet the threshold to smolt at age 0 (Figure 2). In this phase of the study, we measured juvenile growth rate in over 2,600 offspring from three consecutive cohorts and used genetic methods to match them to over 2,400 potential parents of hatchery and natural origin. Second, using data for offspring implanted with passive‐integrated‐transponder (PIT) tags (Prentice, Flagg, & McCutcheon, 1990), we tested the hypothesis that juveniles that grew faster also tended to migrate faster, as would be expected if they were destined to be subyearling smolts. Finally, using the pedigree generated from the parentage analysis we estimated heritability of juvenile growth rate in SRFCS. Results provide additional support for the hypothesis that some evolutionary change in juvenile life history of this threatened population could have accompanied the plastic responses to altered environmental conditions. We also report surprising results for a group of parents that were reared captively for a full year before release as yearling smolts: On average, these parents produced the fastest growing offspring of all! This could represent an example of transgenerational phenotypic plasticity, in which the environment the parent experiences early in its life influences life‐history traits of its offspring. We suggest that SRFCS is an example of a potentially large class of “anthro‐evolutionary” species—those whose evolutionary trajectories have been profoundly shaped by altered selective regimes in human‐dominated landscapes.

## 2 MATERIAL AND METHODS

Snake River fall Chinook salmon are managed as a single, integrated hatchery–natural population, with a small fraction (~10%) of naturally produced fish taken for broodstock each year and large numbers of returning hatchery adults allowed to spawn naturally (Marshall & Small, 2010). Lyons Ferry Hatchery in Washington (LFH) and the Nez Perce Tribal Hatchery in Idaho (NPTH) both propagate SRFCS; LFH releases juveniles as both subyearlings and yearlings, while NPTH only produces subyearlings. Some of the broodstock used at NPTH were released as juveniles from LFH. Because SRFCS are federally listed under the ESA, our experiment had to be implemented within the constraints of research and monitoring projects that were already underway. Empirical data were collected from samples taken from adult carcasses after they were spawned at NPTH and from fin clips taken from juveniles, while they were anesthetized at NPTH for PIT tagging for a separate migration study.

### 2.1 Broodstock sampling and composition

Adult SRFCS were spawned at NPTH over consecutive weeks in October and November, 2007–2009. Adults were either “volunteers” that returned to the hatchery on their own or “transports” that were trapped and trucked from Lower Granite Dam (Figure 1). The number of parents spawned ranged from 574 to 1,064 (Table 1). In 2007–2008, the sex ratio was 1:1 and most families were full siblings. In 2009 there were more females than males, so more half siblings were produced.

**Table 1:** Number of male and female Chinook salmon spawned at Nez Perce Tribal Hatchery 2007–2009

Year	Males	Females	Total
2007	287	287	574
2008	532	532	1064
2009	340	494	834

For each adult, spawn date, fork length (to nearest cm), and sex were recorded, as well as information from coded‐wire tags (CWTs) and PIT tags, when present. Caudal fin tissue collected from postspawning carcasses was dried on filter paper for DNA analysis (LaHood, Miller, Apland, & Ford, 2008). Scales were collected from adult spawners as described by Clutter and Whitesel (1956), and age at smolting was inferred as described by Jerald (1983).

### 2.2 Juvenile life‐history designations of adult spawners

Each spawner was assigned to one of four categories based on its juvenile life history: subyearling smolt (S), yearling smolt (Y), forced‐yearling smolt (FY), and unknown (UNK). Y indicates a fish that volitionally remained over winter in fresh water and subsequently migrated as a yearling; this category included both naturally produced juveniles and hatchery juveniles that were released as subyearlings but did not migrate that year. FY indicates a fish held for a full year at LFH before release, which therefore never had an opportunity to migrate as a subyearling. Spawners with CWT information indicating a yearling release were assigned FY. Fish with regenerated scales and no relevant CWT or PIT information were considered UNK. Direct age validation was possible for some individuals using CWT and/or PIT‐tag information. Based on CWTs, PIT tags, and scale analysis (Connor et al., 2005), origin for each adult was determined as *H* (reared from the egg in a hatchery), *W* (reared in the wild), or unknown.

The distribution of life‐history types in the adult spawners is shown in Table S1.

### 2.3 Juvenile rearing, sampling, and tagging

Fertilized eggs from each family were placed in separate vertical incubation trays. When yolk sacs were absorbed (about 0.5 g), progeny from 8–10 families were combined in 210 ft^3^ fiberglass vats. Fry were sent to acclimation sites when they reached about 2.5 g. Four sites were used in 2007: north and south ponds at the hatchery (NPTH‐N and NPTH‐S), Luke's Gulch (LG), and Cedar Flats (CF) (Figure 1). Two additional sites were added to accommodate larger production in 2008 and 2009: North Lapwai Valley east and west ponds (NLV‐E and NLV‐W) (see Table S2). At each site, fin clips for DNA analysis were taken and fork lengths measured from a random subset of juveniles, while they were anaesthetized for PIT tagging, which occurred in late spring shortly before release at ~60–100 mm length. Sample sizes per site per year averaged *n *=* *199 (range 93–521; Table S3). Because it was not feasible to measure and tag individual fish at the time they emerged as fry, a fixed value of 35 mm (approximate mean size of emergent fry) was used for initial length, and growth rate was calculated as (length at tagging – 35)/*d*, where *d* was elapsed time in days between date of tagging and date of emergence.

### 2.4 Genotyping

Genomic DNA was extracted using the DNeasy Tissue Kit either manually or on a BioRobot 8000 (Qiagen Inc., Valencia, CA, USA). We used eleven microsatellite loci (ten for BY2008; Table S4) that are part of a larger panel used by the Genetic Analysis of Pacific Salmonids (GAPS) Consortium. GAPS loci were chosen for their consistency and reliability across multiple laboratories, and protocols followed Seeb et al. (2007).

For brood year 2007, automated genotype calls were scored independently by two people. Discrepancies were re‐evaluated in the Genotyper file to arrive at a consensus genotype. If discrepancies occurred at more than one locus in an individual, that sample was re‐run for that multiplex set; this occurred at rates of 1%–2%. The 2008 and 2009 brood year samples were scored by a single person. Genotyping error was estimated by repeating a random set of 95 samples for both parents and offspring and comparing the genotypes to the original scores. This produced an estimated error rate of 0.5% per individual per locus. Preliminary analyses of basic population‐genetic parameters (amount of genetic variation; agreement with Hardy–Weinberg expectations) were carried out using FSTAT (Goudet, 2001).

### 2.5 Parentage assignments

For each year of juvenile collections, all spawners from the previous year were considered potential parents. Parentage assignments were carried out using both CERVUS (Kalinowski, Taper, & Marshall, 2007; Marshall, Slate, Kruuk, & Pemberton, 1998) and Colony2 (Jones & Wang, 2010). In CERVUS, using the parent pair with known sex option, an initial simulation was run in which the number of candidate parents, their allele frequencies, and estimates of missing parents, genotype error rate, and the amount of missing data, were used to determine the confidence of each parentage assignment. We used the two‐most‐likely‐parent‐pair option and allowed for missing data at up to two loci. We set the percent‐sampled‐parents parameter at 99% and used the default genotyping error rate of 0.01. Colony2 considers full and half‐sib relationships as well as parent offspring relationships in the likelihood calculations. We used the same values as above for genotyping error and proportion of parents. Assignments were made in CERVUS when the difference in log‐likelihood (LOD score) between the first and second most likely cross was at least 10, and assignments in Colony2 were made when the parental pair was identified with probability >90%. We compared results of the parentage assignments to a matrix of known crosses that were made at the hatchery. We accepted putative assignments when any of the following conditions was met:

- Both programs called the same parent pair, and the cross was in the spawning matrix;
- The cross was not in the matrix, but both putative parents were spawned in the same week, and multiple offspring were assigned to the same cross;
- CERVUS assigned a parent pair and Colony2 assigned one of the same parents, the cross was in the matrix, and multiple offspring were assigned to the cross.
- Colony2 assigned a parent pair and CERVUS did not, but the cross was in the matrix and had multiple offspring assigned to it.

The “cross‐in‐the‐matrix” criterion was relaxed in 2009 because the breeding design was more complicated that year and the spawning matrix proved to be less reliable.

### 2.6 Life‐history modeling

Ideally, juvenile life history of each offspring would be known so it could be compared to the parental life histories. But that requires waiting until progeny return as adults, and those analyses are still ongoing. Instead, we used juvenile growth rate in captivity as a surrogate measure of juvenile life history. We tested whether parental life history was associated with growth rate of juveniles, after accounting for other covariates. A priori*,* we hypothesized that parents with subyearling migrant life histories would produce offspring with faster growth rates.

We used linear models to test this hypothesis and included as covariates the following factors: rearing location (Site), parental brood year (Year* *=* *2007, 2008, 2009), ordinal date of spawning (Spawn), number of days eggs were incubated (Tray), and data on female and male parents: fork length in cm (MFL, FFL) and origin (MO, FO; hatchery, wild, or unknown). Spawn and Tray were included to evaluate effects of seasonal timing of embryonic development and juvenile growth. We also introduced a Site × Year interaction to account for annual variation in unmeasured conditions at the rearing sites. The general model form for growth rate was

(1) $$y_{i}=\beta_{0}+\sum_{k}\beta_{k}x_{k,i}+e_{i}$$

where *y*_*i*_ is the growth rate for individual *i* (*i *=* *1, …, *n*), β_0_ is the intercept, *x*_*k*,*i*_ is the value of explanatory variable *k* for individual *i*, β_*k*_ is the coefficient associated with variable *k*, and the $e_{i}$ are independent random errors normally distributed with mean zero and constant variance.

We first built a multiple regression model based only on the covariate effects and used Akaike's information criterion (AIC) to select the best model that did not include parental life history. We then added the male and/or female life‐history variables to the best covariate model to test hypotheses regarding life histories and to estimate life‐history effects. We also tested an interaction between the male and female life‐history types. Such an interaction would suggest that the contribution of a particular individual to juvenile growth rate would depend on who it mated with. Although the interaction was statistically significant (α* *= 0.05), it was driven by a set of apparently spurious effects produced by combinations of a known life‐history category of one parent and an unknown category for the other parent. This result, along with the fact that we did not have a strong biological justification for including the interaction, led us to drop the life‐history interaction from the analyses. The result was an additive model of life‐history effects.

### 2.7 Migration rate

If our hypothesis that faster‐growing individuals are more likely to migrate directly to sea as subyearlings is true, it follows that these individuals should have faster overall rates of travel downstream after release. Similarly, we expected that slower‐growing individuals would delay or interrupt migration to seek opportunities for further growth and hence have slower overall migration rates and be more likely to overwinter and continue migration as yearlings. We tested this using migration data for fish implanted with PIT tags. Following release as subyearlings, each PIT‐tagged fish had an opportunity for detection at each of seven downstream hydroelectric dams equipped with PIT‐tag detection systems (four on the Snake River and three on the lower Columbia River; Prentice, Flagg, McCutcheon, & Brastow, 1990; Faulkner, Smith, Muir, Marsh, & Williams, 2007). The first dam encountered is Lower Granite Dam on the Snake River (695 km from the ocean), and the last dam encountered is Bonneville Dam on the Columbia River (234 km from the ocean). Because these dams offer multiple routes of passage, and only juvenile bypass systems have detectors, detection probabilities for fall Chinook passing through dams are low. Based on mark‐recapture estimates of detection probabilities at each dam for yearly release cohorts of our study fish from each rearing site, we estimated that on average only 16% of the fish passing any individual dam were detected (range 5%‐32%). Although we could not know the exact timing and duration of migration for each individual fish due to incomplete detection histories, we could calculate an overall migration rate as distance/time, where distance is river km traveled between release site and the last detection location and time is number of days to travel to that location.

We assumed that detection probability was independent of migration rate. This assumption would be violated by fish that delayed migration until winter, when the juvenile detection systems at the dams are turned off. It would also be violated by fish that delayed migration and then died before detection. The result of these violations would be an under‐representation of slow‐migrating fish. Measured growth rate is correlated with length at tagging, and larger juveniles tend to migrate faster, so there is potential for confounding between growth rate and length. As a parallel hypothesis, we tested whether parental life history was associated with migration rate.

We used multiple linear regression models similar to Equation 1 to test our hypotheses, where the natural logarithm of migration rate was a linear function of the set of explanatory variables of interest. We first built a model to predict migration rate using a set of covariates potentially associated with migration rates: categorical variables migration Year, site of release (Site), and last detection location (Detect), and the continuous variables water velocity experience (Vel) and water temperature experience (Temp). We included Year and Site as surrogates for unmeasured sources of variation in migration rate, and we included a Site × Year interaction to allow the site effect to change by year. We included Detect to account for effects of river location not accounted for by the other variables. Water temperature and water velocity can affect migration rates of fall Chinook salmon (Tiffan, Kock, Haskell, Connor, & Steinhorst, 2009; Tiffan et al., 2012). Using data for flow and temperature from the Columbia River DART Web site (http://www.cbr.washington.edu/dart), we calculated Vel and Temp by first calculating day of arrival at each detection location between the last detection and release sites, which was either known directly by intermediate detections or was interpolated based on migration rates between locations. We then calculated average values for velocity and temperature for each fish based on their daily measured values of these variables and the associated number of days spent in each reach. A model‐selection step was then performed to trim any unnecessary covariates. We then added growth rate and/or the parental life‐history variables to see whether the model improved. We used AIC as a measure of predictive ability for all models. We natural log‐transformed the migration rates to account for the fact that migration rates are non‐negative. A summary of explanatory variables used in the regression analyses is in Table S5.

### 2.8 Heritability

A univariate animal model was employed to estimate heritability of growth rate, as implemented in the program WOMBAT (Meyer, 2007). The animal model is a form of general linear mixed model that incorporates as a random factor the breeding value of each individual—an individual's contribution to the trait phenotype in a population, measured as the deviation of its relatives from the population mean. The model provides an unbiased estimate of a trait's genetic and phenotypic variance and heritability (Wilson et al., 2010). The model was fitted using a restricted maximum‐likelihood (REML) algorithm that computes an average information matrix to derive the estimates and their approximate sampling errors for sparse covariance matrices (Johnson & Thompson, 1995). Growth rate was considered a Gaussian trait for the analyses.

The animal model used was of the form

(2) $$y_{i}=\mu +a_{i}+\;\sum_{j}\beta_{j}f_{ij}+\sum_{k}r_{ik}+e_{i}$$

where *y*_*i*_ is the phenotype for trait *y* in individual *i* (*i *=* *1, …, *n*), μ is the population mean, *a*_*i*_ is the random effect of *i*'s breeding value (the contribution of *i* to the distribution of *y* relative to μ, as estimated from the phenotypes of its relatives), *f*_*ij*_ is the value of fixed effect *j* for individual *i*, β_*j*_ is the coefficient associated with fixed effect *j*,* r*_*ik*_ is the value of random effect *k* for individual *i*, and *e*_*i*_ is the residual error term associated with individual *i*. The random effects for individual breeding values follow a multivariate normal distribution with mean zero, where the structure of the covariance matrix depends on the set of pedigrees. Other random effects are also normally distributed with mean zero but are assumed independent. Alternative mixed models incorporated brood year (Year), rearing site, and female (maternal) fork length (FFL) as fixed factors and breeding value as a random factor. Akaike's information criterion corrected for sample size (AIC_c_) was used to evaluate the fit of alternative models to the data, and AIC_c_ values computed by WOMBAT for models with and without the random animal term were compared to determine the statistical significance of estimates.

Heritability of growth rate was estimated as the ratio of the genetic variance (*V*_G_) to the total phenotypic variance (*V*_P_), where *V*_P_ is the sum of *V*_G_ and the residual variance *V*_R_, which includes environmental variance unaccounted for by additional fixed or random effects in the model, nonadditive genetic variance, and error variance (Falconer & Mackay, 1996). Heritability estimates were computed conventionally from the variance of breeding values and the residual variance. Because primarily full‐sibling families were available for the study, the estimates of genetic variance and heritability are likely to be inflated by nonadditive genetic and maternal or common environmental effects and are closer to broad‐sense (*H* ^2^) than to narrow‐sense heritability (*h* ^2^). The number of REML iterations run for each analysis was at least 1,000 with a convergence criterion of change in the log‐likelihood equal to or <0.0001.

## 3 RESULTS

### 3.1 Molecular genetics

#### 3.1.1 Descriptive statistics

Mean heterozygosity was high (ca 0.88–0.9) in both adults and juveniles (Table S6). In three years of adult samples, only one of 32 single‐locus tests (3.1%) showed a significant deviation from expected Hardy–Weinberg proportions (positive *F*_*IS*_, indicating a deficit of heterozygotes; Table S6). In the three years of pooled juvenile samples, seven of 32 tests (21.8%) were significant. The same locus having the significant deviation in the 2009 adults (Omm1080) also had a significant deficit of heterozygotes in all three years of juvenile samples, which suggests that a null allele(s) might be present at this locus. The other deviations in the pooled juvenile collections could be an artifact of combining samples from separate acclimation sites, where largely nonoverlapping sets of families were raised. The site‐specific juvenile samples each included progeny from a relatively small number of families (Table S2). Because a small number of parents tend to produce an excess of heterozygotes (Pudovkin, Zaykin, & Hedgecock, 1996), it is not surprising that these samples showed more loci with significant heterozygote excesses (9) than deficits (5) (Table S7). The 14 significant departures (8.2%) were spread across six different loci, consistent with effects at the level of the samples. Collectively, these results are consistent with a wide range of other genetic studies of Chinook salmon that have concluded that these same loci accurately reflect underlying genetic variation (Narum, Hess, & Matala, 2010; Seeb et al., 2007).

#### 3.1.2 Parentage assignments

Of the 2,472 parents spawned in 2007–2009 (Table 1), all but six (99.8%) were successfully genotyped for all 11 loci; the remainder had poor‐quality DNA or duplicate multilocus genotypes indicating a sampling/labeling error. After applying the criteria described in Methods, we successfully matched 2,670 juveniles (over 800 each year) to both male and female parents (Table 2). For most years and locations, this represented over 90% of the juvenile fin clips that were analyzed (Table S3).

**Table 2:** Number of juvenile Chinook salmon successfully matched to their parents using genetic parentage analysis, by brood year and rearing site (NPTH‐NP and NPTH‐SP, north and south ponds at Nez Perce Tribal Hatchery; CF, Cedar Flats; LG, Luke's Gulch; NLV‐W and NLV‐E, North Lapwai Valley west and east)

Site	2007	2008	2009
NPTH‐NP	184	137	85
NPTH‐SP	200	185	90
LG	240	121	267
CF	238	134	87
NLV‐W	–	181	176
NLV‐E	–	71	174
Total	862	929	879

### 3.2 Life‐history modeling

Estimated juvenile growth rates ranged from 0.260 to 0.763 mm/day (mean* *=* *0.464, *SD *=* *0.067). The best covariate‐only model included all covariates except male and female parental origin (Table 3), neither of which was significant (*p *>* *.2). The effect on growth rate of each of the nonbiological covariates (Site, Year, the Site × Year interaction, Spawn date, and Tray) was highly significant (analysis of variance, Table S8). As expected, we found a highly significant maternal effect, with larger females on average producing faster‐growing offspring, and a smaller, albeit still highly significant, effect of male size (Table S8). In the best‐fitting model, a 10‐cm increase in fork length of a female parent was associated with an estimated increase in mean juvenile growth rate of 0.015 mm/day, while the same increase in fork length of a male parent had an effect only 1/5 as large. After accounting for covariates, we found evidence that growth rate was associated with parental life history (Table 3). The best model included life‐history variables for both parents, although female life history contributed most to the improvement in AIC.

**Table 3:** Results of fitting regression models for juvenile growth rate. full.cov, full set of covariates; best.cov, set of covariates in best model with covariates only; FLH, female life history; MLH, male life history

Model	*np*	Rank	∆AIC	Adj‐*R* ^2^
full.cov	24	5	13.6	.260
best.cov	20	4	9.4	.260
best.cov + MLH	23	3	7.0	.262
best.cov + FLH	23	2	0.9	.263
best.cov + MLH + FLH	26	1	0.0	.264

*np*, number of model parameters; ∆AIC, change in AIC from best‐fitting model; Adj‐*R* ^2^, adjusted *R* ^2^.

**Figure 3:**
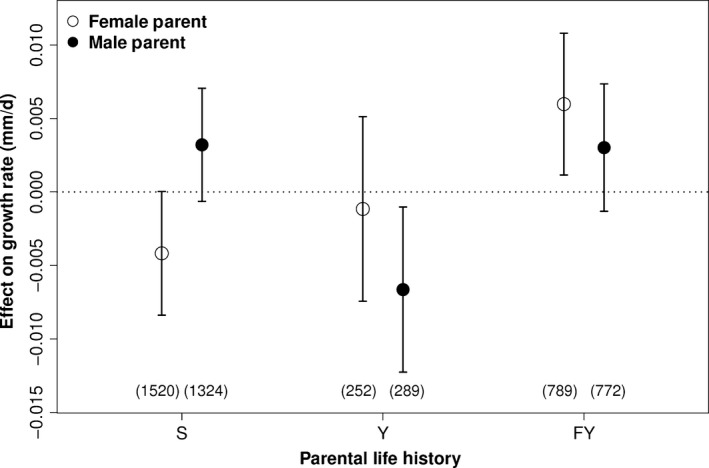
Effect of parental life history (S, subyearling smolt; Y, volitional yearling smolt; FY, forced‐yearling smolt) on predicted growth rate relative to the overall mean growth rate for each parent/sex. Results are for the best model by AIC (see Table 3). Bars represent 95% confidence intervals for predicted effects, and the horizontal dotted line is a point of reference for measuring the magnitude of each effect. Numbers in parentheses are sample sizes for each group. Note that life‐history groupings are not mutually exclusive between parent sexes, and that unknown life histories (for one parent only) are used in estimating effects but are not shown

The parental life‐history effect on growth rate was sex specific (Figure 3). Offspring of S and FY fathers grew at comparable rates, but both groups grew significantly faster than offspring of Y fathers (*p *<* *.02). Offspring with FY mothers grew significantly faster than offspring of S mothers (*p *=* *.0003) and faster (but not significantly) than offspring of Y mothers (*p *=* *.11), while offspring means for the S and Y mothers did not differ significantly.

**Figure 4:**
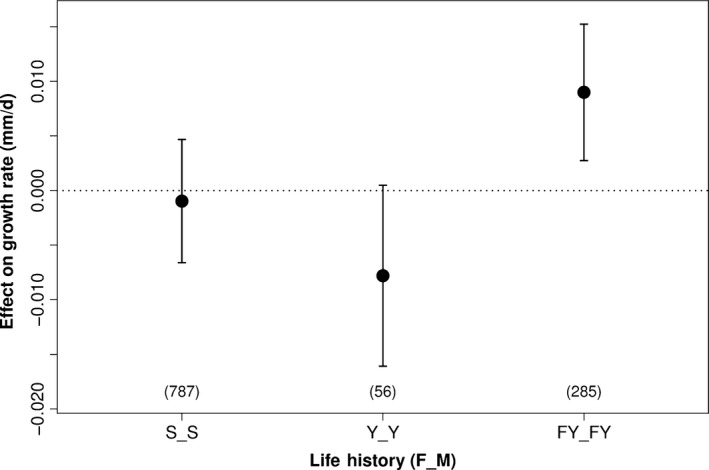
Predicted growth rate for individuals with pure crosses of parental life histories (S, subyearling smolt; Y, volitional yearling smolt; FY, forced‐yearling smolt) from best model by AIC. Bars represent 95% confidence intervals for predicted effects, and the horizontal dotted line is a point of reference for measuring the magnitude of each effect. Note that all possible life‐history crosses go into estimating the relative effects but only results for pure crosses are shown. Numbers in parentheses are sample sizes for each group

Effects of combined parental life histories are most directly assessed by comparing pure crosses. Fish with two S parents grew faster (by 0.0068 mm/day) than those with two Y parents (Figure 4), although the difference was not significant (*p *=* *.21). An unexpected result was that juveniles with two FY parents grew the fastest of all—0.0100 mm/day faster than fish with two S parents (*p *=* *.015) and 0.0168 mm/day faster than those with two yearling parents (*p *=* *.004). Sample size for the YxY crosses was small, which increases uncertainty in the estimates (Figure 4).

### 3.3 Migration rate

Across the three cohorts, 1,041 juveniles (40% of those with growth rate data) were detected at one or more dams after release. Observed migration rates to the last detection location ranged from 1.1 to 58.4 km/day (mean* *=* *12.4, *SD* = 7.4). The best covariate‐only model was the full model with release Site, release Year, Detect, Vel, and Temp. Migration rate was associated with Growth after accounting for the other covariates (Table S9), as evidenced by a decrease in AIC of 19.2 relative to the model with covariates only (Table 4). However, adding life‐history terms to the best covariate model without growth resulted in a poorer fit. The best model overall by AIC included Growth and the female life‐history (FLH) terms, but adding FLH reduced AIC by only 0.5 relative to the model with only Growth, which is a negligible difference. Using the best model, an increase in growth rate of 0.1 mm/day was associated with an estimated increase in median migration rate of 8.7% (95% CI: 4.9%–12.7%).

**Table 4:** Results of fitting regression models for juvenile migration rate. best.cov, set of covariates in best model with covariates only; FLH, female life history; MLH, male life history; Growth, growth rate

Model	*np*	Rank	∆AIC	Adj‐*R* ^2^
best.cov	24	5	19.7	.742
best.cov + MLH	27	7	24.4	.742
best.cov + FLH	27	6	19.8	.743
best.cov + MLH + FLH	30	8	24.5	.743
best.cov + Growth	25	2	0.5	.747
best.cov + Growth + MLH	28	4	5.2	.747
best.cov + Growth + FLH	28	1	0.0	.748
best.cov + Growth + MLH + FLH	31	3	4.5	.748

*np*, number of model parameters; ∆AIC, change in AIC from best‐fitting model; Adj‐*R* ^2^, adjusted *R* ^2^.

Because the best model indicated that Site, Year, and the Site × Year interaction were all significantly associated with Growth, we examined the relationship between growth rate and the natural log of migration rate separately for each site and year (Table 5). We found a positive relationship between growth rate and migration rate at every site in every year. Of the 16 total Site/Year comparisons, 12 (75%) were significant (*p *<* *.05 for a one‐tailed test), and all 12 remained significant after adjustment for multiple testing using the false‐discovery rate (Benjamini & Hochberg, 1995) at α* *=* *0.05.

**Table 5:** Top: correlations between juvenile growth rate and the natural log of migration rate, by site and year. *p <* *.05; **p <* *.01; ***p <* *.001 for a one‐tailed test for a positive correlation. All significant correlations remained significant after applying a false‐discovery rate correction (α* *=* *0.05) for multiple testing. Bottom: sample sizes by site and year. See Figure 1 and Table 2 for site abbreviations

Year	CF	LG	NPTH‐SP	NPTH‐NP	NLV‐W	NLV‐E
2007	0.387***	0.200*	0.308*	0.429***	–	–
2008	0.428**	0.615***	0.308**	0.157	0.075	0.020
2009	0.635***	0.482***	0.322*	0.136	0.268**	0.302**
2007	76	88	56	56	–	–
2008	49	53	68	44	97	68
2009	28	116	39	41	87	75

Although migration rates provide a standardized measure of speed of travel, fish with extremely long travel times can provide a more intuitive indication of individuals that are temporarily stopping or significantly slowing migration. Ten fish (0.96%) had travel times in excess of 100 days from time of release to the last detection location, and five of those had travel times over 200 days (the longest was 309 days). These times were far longer than the 95% quantile value of 58 days at the farthest downstream detection location (Bonneville Dam), which is strong evidence that these fish delayed their migration at some point after release. Half (5) of these holdovers had growth rates in the lowest 5%, and nine of the ten had growth rates in the lowest 50%. Note that these fish provide an estimate of the minimum number of fish to hold over. The true number is greater, since many fish that delay migration are never detected or are only detected at upstream sites before slowing/halting migration.

### 3.4 Heritability

For every scenario we considered, including heritability estimates by Site and Year, including female fork length as a fixed effect always provided the best fit to the data (Table S10). When sites were combined within brood years, only for 2009 was the effect of Site significant. Considering all the data together, the best model included a fixed effect for FFL but not for Site or Year (Table S10).

Broad‐sense heritability estimates for the best‐fitting models each year all fell within a narrow range of 0.733 to 0.804 (Table 6). The 95% confidence intervals of cohort‐specific estimates did overlap, and the lower bound for each yearly estimate was above 0.5. The estimate for all brood years combined (0.775) fell within the range for the individual years; because of a larger sample size, it had a smaller standard error and tighter confidence limits (0.683–0.867).

**Table 6:** Estimates of broad‐sense heritability (*H* ^2^) for growth rate of fall Chinook salmon from each of the best‐fitting models that considered the fixed effects of brood year (BY), female fork length (FFL), and rearing site. Model selection was based on AIC_c_. The random effect of individual breeding value (“animal”) is present in all models. *H* ^2^ estimates are from animal models using a restricted maximum‐likelihood algorithm; all estimates differ significantly (*p *<* *.05) from zero. SE, standard error; CI, confidence interval. See Table S10 for detailed results from all competing models, including for each site each year

Model	Fixed	Random	*H* ^2^	SE (*H* ^2^)	95% CI
BY2007	FFL	Animal	0.733	0.098	0.541–0.925
BY2008	FFL	Animal	0.804	0.078	0.651–0.957
BY2009	Site, FFL	Animal	0.744	0.093	0.562–0.926
All BYs	FFL	Animal	0.775	0.047	0.683–0.867

## 4 DISCUSSION

### 4.1 Adaptation in juvenile life history to anthropogenic habitat change

We found support from three different analyses for the hypothesis that evolution could be partially responsible for the shift in juvenile life history of SRFCS. First, a necessary condition for evolution of any trait is that it be heritable. The animal model provides a flexible way to estimate heritability, and our large sample sizes of juveniles (more than 800 for three consecutive years) lent considerable power to these analyses. Estimates of heritability for juvenile growth rate for individual brood years were all significantly >0.5 and all in the range 0.7–0.8, as was the overall estimate. Because mostly full‐sibling families were produced in all years, these estimates are best interpreted as broad‐sense heritabilities and are likely to be inflated by nonadditive genetic effects and those arising from maternal and common environment sources. In their review of salmonids, Carlson and Seamons (2008) summarized available estimates of heritability for growth and development; these had a median of 0.22 but ranged from near 0.0 to 1.0, depending on the breeding design. Most estimates were from fish held their entire lives in captivity. Few estimates are available for freely migrating Chinook salmon, but Hard (2004) estimated a narrow‐sense heritability of marine growth rate (±SE) for fall Chinook salmon in Puget Sound at 0.31 ± 0.20; a corresponding broad‐sense estimate could be considerably higher. Under an assumption that freshwater and marine growth rates have similar heritabilities, the broad‐sense estimates in the present paper lie within the upper confidence interval for this estimate (*h* ^2^ ~0.70) and imply that our estimates are likely to include substantial nonadditive genetic or environmental sources of variation. Nevertheless, these results indicate that evolution of juvenile growth rate can be expected to occur, provided selective forces favor such evolution (as was demonstrated by Williams et al., 2008 and Hegg, Kennedy, Chittaro, & Zabel, 2013).

Second, a key hypothesis in our study was that juveniles that grow faster are more likely to migrate to sea as subyearlings, as predicted from the threshold model (Figure 2). We tested this by evaluating the relationship between juvenile growth rate and migration rate after release. After accounting for covariates, we found a significantly positive effect of growth rate on migration rate, and within each site in each year we found a positive correlation between growth rate and migration rate (75% of these correlations were significantly positive). Collectively, these data support our assumption that juvenile growth rate is a good indicator of subsequent age at smolting.

Third, the linear modeling of parental life history versus juvenile growth rate provides modest additional support for genetic change. Although the regression models indicate significant associations between juvenile growth rate and parental life history, the adjusted *R* ^2^ values were relatively low (.26 for best model; Table 3), indicating that considerable variation in growth rate remains unexplained by our models. In accordance with our hypothesis, male parents who were S migrants produced offspring that grew significantly faster than male parents who were Y migrants. However, female parents who were S migrants had offspring with nonsignificantly lower growth rates than female parents who were Y migrants. On average, offspring with both parents having the S phenotype grew faster than offspring with both parents having the Y phenotype, but the difference was not statistically significant. The latter analyses were constrained by small sample sizes of offspring for which both parents were yearling migrants (Figure 4).

These results do not prove that evolution of smolt age has occurred in SRFCS—only that all the ingredients for evolution appear to be present. Environmental conditions, especially water temperature and flow, clearly can have a large influence on smolt age, and it seems likely that human manipulation of flow regimes has affected the incidence of yearling smolts in SRFCS (Hegg et al., 2013). It is noteworthy, however, that environmental variability experienced by the population in its historical habitat apparently was not sufficient to produce any detectable fraction of yearling migrants. In fact, essentially 100% expression of the subyearling life‐history trait was an important factor that led to the conclusion that SRFCS are a separate ESA “species” from Snake River spring‐summer Chinook salmon, which produce essentially 100% yearling smolts (Waples, Teel, Myers, & Marshall, 2004; Waples et al., 1991).

**Figure 5:**
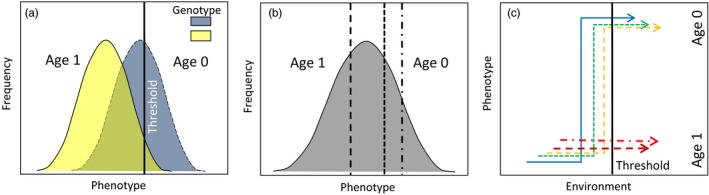
Three versions of a threshold model for expression of a life‐history trait. (a) The phenotype, determined by a combination of genetic and environmental factors, is compared to a fixed threshold (solid vertical line) to determine whether smolt migration will occur at age 0 or age 1 (after Falconer, 1965; Thorpe, Mangel, Metcalfe, & Huntingford, 1998). (b) Different individuals have different genetically determined thresholds (dashed vertical lines) (see Tomkins & Hazel, 2007). (c) Different individuals have different genetically determined reaction norms, which are compared with a fixed environmental threshold to determine trait expression (see Roff, 1996). In (a) the phenotype determines the trait, given the fixed threshold; in (b) individuals with the same phenotype can express different traits, depending on their genetically based thresholds; in (c), individuals experiencing the same environment can express different traits due to plasticity resulting from individual variation in genetically determined reaction norms. Panel (c) depicts the possibility that some individuals will never express the age 0 phenotype due to their inherent reaction norms

Several variations of the threshold model illustrated in Figure 2 have been proposed; they differ in the relative importance of genetic and environmental factors in determining the individual phenotype and/or the threshold (Figure 5). In one version of this model (Figure 5a), the threshold is fixed, while the phenotype of each individual reflects a combination of plastic responses to environmental conditions and genetically based traits such as growth rate or feeding efficiency. In other variations of the model, individuals can have different genetically based thresholds (Figure 5b) or genetically based reaction norms (Figure 5c). The most likely mechanisms to produce genetic change in smolt age are evolution of juvenile growth rate (or correlated traits such as feeding behavior and metabolic rate), as studied here with reference to the model in Figure 5a, evolution of the threshold to trigger smoltification and downstream migratory behavior (as in Figure 5b), or evolution of the reaction norm for expressing the smolt behavior (as in Figure 5c). Our data are not sufficient to distinguish among these different scenarios, but all could lead to largely the same conclusions about consequences of evolutionary change for conservation and management. Recent evolution of the reaction norm for smolting has been demonstrated in a closely related species, rainbow trout/steelhead (*Oncorhynchus mykiss*; Phillis et al., 2016).

If adaptation to the current hydropower configuration of their habitat has been at least partially responsible for the observed increase in yearling smolts in SRFCS, what might be the consequences if the four dams on the lower Snake River (numbers 1–4 in Figure 1) are removed? This is not as far‐fetched an idea as it might appear, as state and tribal agencies and other groups have advocated for this for over two decades (USACE 2010), and a recent court ruling asked NMFS to consider this option more fully (NWF v NMFS 2016). Furthermore, the recent removal of two large, century‐old dams on the Elwha River in Washington State to restore passage for salmon shows that such events are possible (East et al., 2015). The four lower Snake River dams are combined earth and concrete structures, so the dams could be bypassed by moving the large earthen berms to the side or removing them (USACE 2010). The result would be a more‐or‐less free‐flowing stretch of river from the mouth of the Snake River to Hells Canyon Dam, which would greatly expand current spawning and rearing habitat, as well as eliminate passage mortality at those four dams. This strategy, however, would also eliminate four reservoirs that currently serve as convenient overwintering habitat for juveniles that do not migrate to sea as subyearlings. It is possible that one reason the yearling life history was not documented in the historic SRFCS population was that juveniles that did not smolt at age 0+ had few or no viable habitats for overwintering. If that scenario were replicated in the lower Snake River, but the population had at least partially committed (through adaptation) to a yearling life history, the consequences could reduce or potentially eliminate survival benefits from removing the dams. This suggests that if any or all of the lower Snake River dams are removed, it should be implemented in an adaptive management framework that includes careful monitoring of response by SRFCS. It would be important to monitor not only traditional metrics like survival and productivity but also changes to juvenile and adult life history. More generally, the best way to maximize the ability of the population to robustly respond to unpredictable conditions in the future is to take management actions that are consistent with maintaining genetic variation for expression of key life‐history traits.

### 4.2 Effects of captive propagation on life history

A major surprise in our study was the finding that parents that were held in the hatchery for a full year and released as yearling smolts (FY phenotype) produced the fastest growing offspring of all. This was true for female parents (Figure 3; male FY parents produced offspring that grew faster than Y parents but not S parents) and for crosses in which both parents were FY (Figure 4). Is it possible that the higher growth rate of progeny of FY parents reflects rapid domestication (e.g., Christie, Marine, French, & Blouin, 2012)? We cannot rule this out entirely, but it seems unlikely. Reisenbichler, Rubin, Wetzel, and Phelps (2004) proposed a mechanism for rapid domestication of steelhead: Juveniles that did not grow fast enough in the hatchery to reach about 150–160 mm at one year of age did not survive well after release. However, whereas a steelhead hatchery has to speed up growth to produce fish that will smolt at age 1 (smolting at age 2 or older is the norm in most natural populations), the forced‐yearling strategy for SRFCS works in the opposite direction, by delaying smolt age by one year from the pattern in natural populations. Furthermore, juveniles raised as yearlings at LFH are randomly chosen each year and do not represent a separate lineage. Therefore, we would not expect the FY strategy to select for parents that produce faster‐growing offspring.

We think it is more likely that this result represents a kind of cross‐generational phenotypic plasticity, whereby the early rearing environment of the parent affects the phenotype of the offspring. Because the observed pattern works in the opposite direction to a typical maternal effect associated with egg size, this potentially could represent effects of hatchery‐induced changes in (i) maternal RNAs present in the egg (Pelegri, 2003) and/or (ii) environmentally induced epigenetic changes in the germline (Gilbert & Epel, 2009) that affect offspring phenotype. These are intriguing areas of research that merit further exploration. Studies in Atlantic salmon have shown that parental history affects offspring phenotype (Burton, McKelvey, Stewart, Armstrong, & Metcalfe, 2013; Van Leeuwen et al., 2016). A recent comparison of offspring from interbreeding between single‐generation hatchery and wild steelhead demonstrated differences in expression of genes related to growth and metabolism at the button up fry stage (Christie, Marine, Fox, French, & Blouin, 2016), which could be due to heritable genetic or epigenetic mechanisms. Other studies have shown that transgenerational phenotypic plasticity can be adaptive in plants (Galloway & Etterson, 2007) and animals (Richter‐Boix, Orizaola, & Laurila, 2014), provided the maternal environment accurately reflects the environment the offspring will encounter. In salmon hatcheries, however, the rearing environment differs dramatically in many ways from that experienced in the wild, which suggests that transgenerational phenotypic plasticity could easily be maladaptive for sustainability of natural populations.

It is important to note that even if the offspring growth rate pattern we observed for FY parents represents phenotypic plasticity, it still can have long‐lasting effects on the population. First, epigenetic modifications to the genome can persist across multiple generations (Herman, Spencer, Donohue, & Sultan, 2014). Second, phenotypes that are altered by plasticity can affect a wide range of ecological interactions, which in turn change the selective regimes experienced by the focal species and hence can lead to evolution (Fordyce, 2006). Juvenile growth rate and age at smoltification play such major roles in the life history of salmonids that plastic effects on this trait can be expected to have substantial ecoevolutionary consequences (Berejikian et al., 2017). Clearly, much remains to be learned about this crucial aspect of captive‐wild systems. The extent of our ignorance about long‐term consequences argues for considerable caution in using captive propagation to manipulate a species’ life history, even when (or perhaps especially when) short‐term demographic benefits can be demonstrated, as in the case of SRFCS.

Future research might focus on common garden experiments of SRFCS involving different temperature regimes during critical developmental stages to assess the degree of plasticity in growth rate and age at smoltification, coupled with genomic and epigenetic characterization of the divergent migrant life histories. This would be a novel opportunity to combine the genomic, epigenetic, and quantitative genetic assessment of this type of life‐history variation in response to one or more environmental variables. Additional insights regarding effects of climate change could be gained by evaluating whether other environmental‐response traits (such as temperature tolerance and disease resistance) differ among life‐history types. Manipulative experiments like these can be difficult or impossible to implement on federally protected species, particularly when (as is the case for SRFCS) every aspect of their management is subject to court supervision as part of the US v Oregon decision regarding treaty fishing rights for Native Americans. Fortunately, a more abundant population of fall Chinook salmon with similar life history exists in the upper Columbia River (Myers et al., 1998), and this potentially could be used as a surrogate for experimental research.

### 4.3 Anthro‐evolutionary species

Selective regimes experienced by SRFCS have changed dramatically in many other ways that might promote adaptive responses, so our evaluations of evolution of smolt age are just the tip of a large iceberg. As with an iceberg, the fraction of environmental changes whose evolutionary consequences have begun to be evaluated (primarily effects of harvest and artificial propagation) is small compared to the vast unexplored regions that are difficult to recognize and attract scant attention. Scott, Goble, Haines, Wiens, and Neel (2010) have argued that a large fraction of protected species in the US (and potentially elsewhere) should be considered “conservation reliant” because their natural habitats have been so altered by humans that constant (perhaps indefinite) human intervention is needed to fend off extinction. What has received little attention is the fact that in the human‐altered habitats of these conservation‐reliant species, selective regimes have also been changed dramatically in ways we are just beginning to understand. Perhaps these species merit a new term—we suggest “anthro‐evolutionary species”—to emphasize the role human modifications to habitats have played in their recent evolutionary histories. Humans have also profoundly affected evolution of abundant, commensal species such as rats and cockroaches, but we primarily call attention to conservation‐reliant species whose evolutionary trajectories have been altered as a direct consequence of being conservation reliant. It follows that, over time, it will become less likely that such species will have the capacity to be naturally self‐sustaining, even if their habitats are eventually restored to something like their historic conditions. This issue has been raised with respect to captive rearing and domestication (Fraser, 2008; McPhee, 2004; Williams & Hoffman, 2009) but appears to be a much more general phenomenon that merits greater attention than it has received to date.

## DATA ARCHIVING STATEMENT

Genetic and phenotypic data collected as part of this study are archived in Dryad Digital Repository: https://doi.org/10.5061/dryad.rc3cp.
