# Supplementary material for: Human‐mediated evolution in a threatened species? Juvenile life‐history changes in Snake River salmon
Source: Evol Appl. 2017 May 19;10(7):667–81. doi: 10.1111/eva.12468 (PMC5511361; doi:10.1111/eva.12468)
Supplement: Supplementary file 1 [file EVA-10-667-s001.pdf]

Supporting Information for:

Human-mediated evolution in a threatened species? Juvenile life-history changes in Snake River salmon

Table S1. Distribution of juvenile life history types among adult spawners, 2007-2009. S = subyearling smolt; Y = yearling smolt; FY = forced yearling smolt. 'Wild' and 'Hatchery' indicate whether the fish was reared in a hatchery or the product of natural spawning.

		Females					Males				
		S	Y	FY	Unk	Total	S	Y	FY	Unk	Total
**2007**	**Wild**	28	29	0	0	57	20	17	0	0	37
	**Hatchery**	84	17	109	4	214	81	29	115	3	228
	**Unknown**	0	0	0	16	16	0	0	0	22	22
	**Total**	112	46	109	20	287	101	46	115	25	287
	**Fraction**	0.39	0.16	0.38	0.07		0.35	0.16	0.40	0.09	
**2008**	**Wild**	21	30	0	0	51	11	20	0	0	31
	**Hatchery**	309	11	138	6	464	399	19	70	10	498
	**Unknown**	0	0	0	17	17	0	0	0	3	3
	**Total**	330	41	138	23	532	410	39	70	13	532
	**Fraction**	0.62	0.08	0.26	0.04		0.77	0.07	0.13	0.02	
**2009**	**Wild**	6	14	0	0	20	5	19	0	0	24
	**Hatchery**	249	14	191	9	463	98	15	147	20	280
	**Unknown**	0	0	0	10	10	0	0	0	32	32
	**Total**	255	28	191	19	494	103	34	147	52	336
	**Fraction**	0.52	0.06	0.39	0.04		0.31	0.10	0.44	0.15	

Table S2. Number of families of juveniles reared at each acclimation site each year. In some cases families were split among two sites, and in two cases entire families were exported to sites outside our study design.

							Split families							
	**NPTH-N**	**NPTH-S**	**LG**	**CF**	**NLV-E**	**NLV-W**	**NPTH-N+S**	**NPTH-S+CF**	**LG+CF**	**NLV-E+W**	**NLV+NPTH**	**Unknown**	**Exported[1]**	**Total**
**2007**	44	66	30	18	-	-	-	11	8	-	1	-	109	287
**2008**	60	128	73	69	-	-	-	-	-	202	-	-	-	532
**2009**	-	-	50	33	83	67	156	-	33	12	-	17	43	494

[1]Reared at a site outside of our experimental design

Table S3. Number of juveniles assayed for DNA data, by year and acclimation site.

	NPTH-N	NPTH-S	LG	CF	NLV-E	NLV-W	Total
**2007**	521[1]	209	242	238	-	-	1210[1]
**2008**	141	237	140	141	188	188	1035
**2009**	94	94	284	93	188	189	942

[1] Includes >200 juveniles who matched to parents spawned at Lyons Ferry Hatchery

Table S4. Microsatellite loci used in the parentage analysis.

Locus	Allele Range	Reference
Ogo2	204-248	Olsen et al. 1998
Ogo4	185-341	Olsen et al. 1998
Ots211	193-315	Grieg et al. 2003
Ots212	185-341	Grieg et al. 2003
Oki100	163-343	DFO unpublished
Ots3M	125-153	Grieg and Banks 1999
Omm1080	163-367	Rexroad et al. 2001
Ots213	177-345	Grieg et al. 2003
Ots201b	140-316	OSU unpublished
Ssa408	184-304	Cairney et al. 2000
Ots2M	130-178	Grieg and Banks 1999

Cairney, M, J. B. Taggart, and B . Hoyheim. 2000. Atlantic salmon (Sdmo salar L.) and cross-species amplification in others salmonid. Molecular Ecology 9:2175-2178.

Greig, C. A., and M. A. Banks. 1999. Five multiplexed microsatellite loci for rapid response run identification of California'es endangered winter Chinook salmon. Animal Genetics 30(4):318-320.

Greig, C., D. P. Jacobson, and M.A. Banks 2003. New tetranucleotide microsatellites for fine-scale discrimination among endangered Chinooks salmon (*Oncorhynchus tshawytscha*). Molecular Ecology Notes 3:376-379.

Olsen, J. B., P. Bentzen, and J. E. Seeb 1998. Characterization of seven microsatellite loci derived from pink salmon. Molecular Ecology 7:1083-1090.

Rexroad, C. E., III, R. L. Coleman, A.M. Martin, W. K. Hershberger and J. Killefer 2001. Thirty-five polymorphic microsatellite markers for rainbow trout (*Oncorhynchus mykiss*). Animal Genetics 32:317-319.

Table S5. Explanatory variables used in regression models

**Variable**	**Code**	**Type**	**Models**
year (brood or migration)	Year	categorical (3 levels)	growth, migration
site (rearing or release)	Site	categorical (4-6 levels)	growth, migration
female life history	FLH	categorical (4 levels)	growth, migration
male life history	MLH	categorical (4 levels)	growth, migration
growth rate (mm/d)	Grow	continuous	growth, migration
female origin	FO	categorical (3 levels)	growth
male origin	MO	categorical (3 levels)	growth
female fork length (cm)	FFL	continuous	growth
male fork length (cm)	MFL	continuous	growth
spawn day	Spawn	continuous	growth
days in tray	Tray	continuous	growth
last detection location	Detect	categorical (7 levels)	migration
water temp (°C)	Temp	continuous	migration
water velocity (km/d)	Vel	continuous	migration

Table S6. Population genetic summary statistics each year for all adults and their juvenile offspring. N is the number of individuals scored at each locus; H is gene diversity; Fis is the inbreeding coefficient. Significance levels for 2-tailed test of H_0, Fis = 0: * (P < 0.05); ** (P < 0.01). Exc (Def) = number of significant single-locus tests with excess (deficit) of heterozygotes.

		Ogo2	Ogo4	Oki100	Ots211	Ots212	Ots3M	Omm 1080	Ots201b	Ots213	Ots2M	Ssa408	Mean	Exc	Def
**Adults**	N	576	576	576	576	576	576	576	576	575	575	576	576		
2007	H	0.858	0.745	0.953	0.936	0.929	0.773	0.962	0.916	0.946	0.845	0.923	0.890		
	Fis	0.013	-0.026	0.005	0.002	-0.013	-0.016	0.007	0.015	0.009	0.006	-0.001	0.001	0	0
	N	1079	1080	1076	1081	1083	-	1074	1081	1080	1082	1079	1080		
2008	H	0.859	0.739	0.950	0.936	0.929	-	0.964	0.924	0.945	0.846	0.922	0.901		
	Fis	0.007	-0.005	0.010	-0.003	-0.016	-	0.01	0.006	0.001	0.006	-0.006	0.001	0	0
	N	831	834	833	832	832	829	832	832	832	833	832	832		
2009	H	0.862	0.763	0.952	0.932	0.945	0.766	0.966	0.929	0.949	0.828	0.917	0.892		
	Fis	0.024	0.023	0.018	-0.024	-0.010	0.020	0.022*	-0.002	-0.001	0.024	0.018	0.010	0	1
Mean	Fis	0.015	-0.003	0.011	-0.008	-0.013	0.002	0.013	0.006	0.003	0.012	0.004			
**Juveniles**	N	1186	1100	1160	1086	1171	1194	1174	1179	1187	1190	1190	1165		
2007	H	0.856	0.729	0.954	0.934	0.917	0.758	0.967	0.912	0.949	0.841	0.926	0.886		
	Fis	0.017	0	-0.007	-0.016	-0.003	-0.033*	0.02**	-0.019*	0.023	0.004	-0.003	-0.001	2	1
	N	961	983	991	989	1012	-	958	989	996	995	1000	987		
2008	H	0.853	0.733	0.952	0.931	0.926	-	0.964	0.920	0.947	0.852	0.922	0.900		
	Fis	0.008	0.009	0.005	-0.013	-0.005	-	0.027**	0.010	0.010	0.001	0.010	0.006	0	1
	N	849	899	906	910	913	895	854	920	916	914	909	899		
2009	H	0.871	0.751	0.949	0.929	0.930	0.765	0.967	0.927	0.948	0.841	0.918	0.891		
	Fis	0.009	0.001	0.013	0.030**	0.005	-0.014	0.023*	0.010	-0.004	0.032*	0.006	0.011	0	3
Mean	Fis	0.011	0.003	0.004	0.000	-0.001	0.010	0.023	0.000	0.010	0.012	0.004			
	Excess	0	0	0	0	0	1	0	1	0	0	0		2	
	Deficit	0	0	0	1	0	0	4	0	0	1	0			6

Table S7. Population genetic summary statistics each year for juvenile samples, by rearing site. N is the number of individuals scored at each locus; H is gene diversity; Fis is the inbreeding coefficient. Significance levels for 2-tailed test of H_0, Fis = 0: * (P < 0.05); ** (P < 0.01). Exc (Def) = number of significant single-locus tests with excess (deficit) of heterozygotes.

	Site		Ogo2	Ogo4	Oki100	Ots211	Ots212	Ots3M	Omm 1080	Ots201b	Ots213	Ots2M	Ssa408	Mean	Exc	Def
	NP	N	498	411	481	400	485	505	490	493	504	509	501	480		
		H	0.84	0.719	0.947	0.933	0.909	0.74	0.965	0.901	0.945	0.839	0.92	0.878		
		Fis	-0.004	0.026	-0.001	-0.003	0	-0.033	0.021	-0.026	0.036**	0.014	0.015	-0.008	0	1
	SP	N	209	209	203	207	207	209	204	206	206	202	209	206		
		H	0.867	0.764	0.953	0.932	0.92	0.76	0.965	0.921	0.947	0.82	0.915	0.888		
**2007**		Fis	0.001	0.029	-0.024	-0.005	-0.060**	-0.115	0.019	0.009	0.021	x	-0.041*	0.013	2	0
	LG	N	242	242	240	241	242	242	242	242	241	241	242	242		
		H	0.884	0.733	0.953	0.927	0.919	0.761	0.96	0.905	0.939	0.832	0.935	0.886		
		Fis	0.069	-0.115**	-0.040**	-0.037	0.027	0.029	0.018	-0.034	0.015	-0.054	-0.038	0.005	2	0
		N	237	238	236	238	237	238	238	238	236	238	238	237		
	CF	H	0.825	0.704	0.945	0.928	0.906	0.779	0.958	0.915	0.951	0.861	0.915	0.881		
		Fis	-0.005	0.030	0.004	-0.042*	-0.007	-0.043	-0.003	-0.032	-0.011	0.028	0.001	-0.013	1	0
	NLVW	N	225	233	232	233	232	-	222	229	231	227	228	229		
		H	0.85	0.719	0.955	0.931	0.925	-	0.966	0.912	0.94	0.841	0.918	0.896		
		Fis	0.060	0.033	0.015	-0.004	-0.011	-	0.013	-0.027	-0.006	-0.002	0.002	0.006	0	0
	NLVE	N	137	139	138	139	138	-	133	132	134	134	132	136		
		H	0.838	0.744	0.949	0.931	0.924	-	0.959	0.929	0.935	0.857	0.918	0.898		
		Fis	0.028	-0.015	0.003	-0.064**	0.003	-	0.03	0.008	0.010	0.010	0.021	-0.016	1	0
	NP	N	141	137	141	132	141	-	130	138	141	129	137	137		
		H	0.837	0.694	0.95	0.93	0.938	-	0.958	0.905	0.953	0.846	0.928	0.894		
**2008**		Fis	0.002	0.038	0.017	0.014	-0.046**	-	0.077	-0.001	0.008	-0.007	0.025	0.003	1	0
	SP	Na	195	201	213	215	226	-	201	219	219	232	231	215		
		H	0.863	0.77	0.955	0.939	0.922	-	0.965	0.921	0.948	0.856	0.919	0.906		
		Fis	-0.017	-0.063	-0.001	0.031	-0.006	-	0.021	0.015	0.01	-0.018	0.001	0.006	0	0
	LG	N	135	137	132	134	136	-	134	135	134	135	134	135		
		H	0.858	0.703	0.941	0.928	0.911	-	0.956	0.908	0.938	0.854	0.914	0.891		
		Fis	-0.044	-0.140*	-0.012	-0.056	0.057*	-	-0.001	0.028	0.011	-0.037	0.001	0.013	1	1
	CF	Na	128	136	135	136	139	-	138	136	137	138	138	136		
		H	0.846	0.729	0.944	0.891	0.923	-	0.955	0.923	0.945	0.846	0.914	0.892		
		Fis	-0.019	0.160**	-0.015	-0.013	-0.016	-	-0.022	0.021	0.006	0.037	-0.037	-0.001	0	1

Table S7, continued.

	Site		Ogo2	Ogo4	Oki100	Ots211	Ots212	Ots3M	Omm 1080	Ots201b	Ots213	Ots2M	Ssa408	Mean	Exc	Def
	NLVW	N	153	169	175	178	181	176	183	185	185	184	185	178		
		H	0.866	0.712	0.948	0.924	0.937	0.77	0.968	0.913	0.942	0.85	0.915	0.886		
		Fis	0.011	-0.030	-0.037*	0.021	0.015	0.011	0.046	0.035	0.019	0.003	0.049	0.010	1	0
	NLVE	N	179	174	179	178	180	175	181	180	181	182	172	178		
		H	0.861	0.777	0.948	0.921	0.934	0.787	0.965	0.926	0.95	0.839	0.905	0.892		
		Fis	-0.039	-0.028	0.010	0.042	-0.023	0.035	0.010	-0.014	-0.007	0.051	-0.015	0.001	0	0
	NP	Na	58	93	92	93	93	89	81	88	87	88	88	86		
		H	0.861	0.752	0.944	0.942	0.936	0.754	0.956	0.939	0.943	0.826	0.924	0.889		
**2009**		Fis	0.018	0.070	0.068	-0.005	0.012	0.031	-0.020	0.020	-0.012	0.023	0.028	0.014	0	0
	SP	N	91	92	90	91	93	92	64	93	90	90	91	89		
		H	0.863	0.71	0.934	0.938	0.907	0.718	0.959	0.937	0.943	0.808	0.923	0.876		
		Fis	-0.031	0.004	0.013	-0.008	-0.019	0.001	-0.027	0.013	-0.049	0.161	0.023	0.002	0	0
	LG	N	280	281	281	280	279	273	270	281	280	279	280	279		
		H	0.875	0.761	0.948	0.923	0.925	0.764	0.964	0.925	0.945	0.841	0.92	0.890		
		Fis	0.037	0.018	-0.006	0.013	0.008	-0.06	0.028	-0.004	-0.009	0.003	-0.021	0.020	0	0
	CF	N	88	90	89	90	87	90	75	93	93	91	93	89		
		H	0.861	0.756	0.936	0.919	0.919	0.768	0.967	0.912	0.94	0.829	0.918	0.884		
		Fis	-0.003	-0.043	0.087**	0.117**	0.025	-0.100	0.035	0.010	-0.018	-0.021	-0.007	0.006	0	2
		Excess	0	2	2	2	2	0	0	0	0	0	1		9	
		Deficit	0	1	1	1	1	0	0	0	1	0	0			5

Table S8. Analysis of variance table for variables from the regression model for growth rate as a function of male and female life history and best set of other covariates. Variable descriptions are given in Table S5.

**Variable**	**df**	**Sum of Sq.**	**F-value**	**Pr(> F)**
Site	5	0.3213	19.3	< 0.0001
Year	2	0.1115	16.7	< 0.0001
Site x Year	8	0.4992	18.7	< 0.0001
Spawn	1	0.1124	33.7	< 0.0001
Tray	1	0.0564	16.9	< 0.0001
FFL	1	0.3698	111.1	< 0.0001
MFL	1	0.0256	7.7	0.0056
FLH	3	0.0429	4.3	0.0049
MLH	3	0.0227	2.3	0.0784
Residuals	2,644			

Table S9. Analysis of variance for variables from the regression model for natural log of migration rate as a function of growth rate and male and female life history and other covariates. Variable descriptions are given in Table S5.

**Variable**	**df**	**Sum of Sq.**	**F-value**	**Pr(> F)**
Site	5	156.2	294.2	< 0.0001
Year	2	18.7	88.1	< 0.0001
Site x Year	8	29.5	34.7	< 0.0001
Detect	6	29.5	46.3	< 0.0001
Vel	1	68.0	640.7	< 0.0001
Temp	1	0.9	8.4	0.0039
Grow	1	2.3	21.5	< 0.0001
FLH	3	0.7	2.1	0.0928
MLH	3	0.2	0.5	0.7000
Residuals	1,010			

Table S10. Estimates of heritability (H^2) for growth rate of fall chinook salmon. Results are shown for each of the models that considered the fixed effects of brood year (BY), female fork length (FFL), and rearing site (see Figure 1). The random effect of individual breeding value ('animal') is present in all models. H^2 estimates are from animal models using a restricted maximum likelihood algorithm; significant ($P < 0.05$) estimates are in italics. V_G, genetic variance; V_R, residual variance; V_P, phenotypic variance; SE, standard error; AIC_c, Akaike's Information Criterion corrected for sample size. For each comparison, the best model by AIC_c is indicated in **bold**.

Model	Fixed	Random	V_G	SE(V_G)	V_R	SE(V_R)	V_P	SE(V_P)	H^2	SE(H^2)	AIC_c
BY2007 NPTH-NP	None	animal	1.966E-03	8.393E-04	1.374E-03	5.623E-04	3.340E-03	4.407E-04	0.589	0.196	443.740
**BY2007 NPTH-NP**	**FFL**	**animal**	**2.993E-03**	**1.895E-03**	**1.000E-03**	**1.050E-03**	**3.993E-03**	**9.437E-04**	**0.750**	**0.313**	**376.433**
BY2007 NPTH-SP	None	animal	2.051E-03	6.522E-04	1.000E-03	5.169E-04	3.051E-03	3.591E-04	0.672	0.177	510.586
**BY2007 NPTH-SP**	**FFL**	**animal**	**2.065E-03**	**9.222E-04**	**1.000E-03**	**6.209E-04**	**3.065E-03**	**4.829E-04**	**0.674**	**0.227**	**422.796**
BY2007 LG	None	animal	2.439E-03	7.723E-04	1.000E-03	4.745E-04	3.439E-03	4.293E-04	0.709	0.158	597.985
**BY2007 LG**	**FFL**	**animal**	**1.225E-03**	**7.344E-04**	**1.349E-03**	**4.332E-04**	**2.574E-03**	**3.980E-04**	**0.476**	**0.224**	**531.819**
BY2007 CF	None	animal	1.872E-03	6.178E-04	1.000E-03	4.120E-04	2.872E-03	3.521E-04	0.652	0.162	622.973
**BY2007 CF**	**FFL**	**animal**	**1.000E-03**	**8.000E-04**	**1.000E-03**	**4.592E-04**	**2.000E-03**	**3.980E-04**	**0.500**	**0.311**	**546.050**
BY2007	None	animal	2.780E-03	4.691E-04	1.000E-03	2.950E-04	3.780E-03	2.539E-04	0.735	0.088	2141.912
BY2007	Site	animal	1.824E-03	2.954E-04	1.000E-03	1.958E-04	2.824E-03	1.659E-04	0.646	0.079	2162.818
**BY2007**	**FFL**	**animal**	**2.740E-03**	**5.292E-04**	**1.000E-03**	**3.186E-04**	**3.740E-03**	**2.815E-04**	**0.733**	**0.098**	**2012.755**
BY2007	Site, FFL	animal	1.996E-03	4.015E-04	1.000E-03	2.455E-04	2.996E-03	2.142E-04	0.666	0.097	2035.109
BY2008 NPTH-NP	None	animal	7.493E-03	2.737E-03	1.000E-03	2.200E-03	8.493E-03	1.220E-03	0.882	0.263	387.045
**BY2008 NPTH-NP**	**FFL**	**animal**	**3.481E-03**	**1.198E-03**	**1.000E-03**	**9.494E-04**	**4.481E-03**	**5.712E-04**	**0.777**	**0.219**	**330.898**
BY2008 NPTH-SP	None	animal	2.544E-03	9.097E-04	1.000E-03	7.448E-04	3.544E-03	4.452E-04	0.718	0.217	336.122
**BY2008 NPTH-SP**	**FFL**	**animal**	**2.928E-03**	**1.198E-03**	**1.000E-03**	**9.164E-04**	**3.928E-03**	**5.742E-04**	**0.745**	**0.245**	**254.120**
BY2008 LG	None	animal	2.083E-03	8.981E-04	1.000E-03	6.673E-04	3.083E-03	4.657E-04	0.676	0.233	301.422
**BY2008 LG**	**FFL**	**animal**	**2.158E-03**	**1.349E-03**	**1.000E-03**	**8.619E-04**	**3.158E-03**	**6.667E-04**	**0.683**	**0.315**	**234.228**
BY2008 CF	None	animal	1.596E-03	7.299E-04	1.311E-03	5.668E-04	2.907E-03	3.890E-04	0.549	0.212	327.375
**BY2008 CF**	**FFL**	**animal**	**1.706E-03**	**9.085E-04**	**1.181E-03**	**6.151E-04**	**2.887E-03**	**4.757E-04**	**0.591**	**0.246**	**271.226**
BY2008 NLV-E	None	animal	3.539E-03	1.066E-03	1.000E-03	7.427E-04	4.539E-03	5.633E-04	0.780	0.174	388.817
**BY2008 NLV-E**	**FFL**	**animal**	**3.590E-03**	**1.510E-03**	**1.000E-03**	**9.545E-04**	**4.590E-03**	**7.418E-04**	**0.782**	**0.230**	**308.926**
BY2008 NLV-W	None	animal	6.102E-03	1.903E-03	1.000E-03	1.449E-03	7.102E-03	9.638E-04	0.859	0.209	401.284
**BY2008 NLV-W**	**FFL**	**animal**	**3.648E-03**	**1.203E-03**	**1.000E-03**	**8.946E-03**	**4.648E-03**	**5.882E-04**	**0.785**	**0.202**	**341.444**

Table S10, Continued.

Model	Fixed	Random	V_G	$SE(V_G)$	V_R	$SE(V_R)$	V_P	$SE(V_P)$	H^2	$SE(H^2)$	AIC_c
BY2008	None	animal	4.774E-03	5.587E-04	1.000E-03	4.366E-04	5.774E-03	2.980E-04	0.827	0.077	2068.530
BY2008	Site	animal	3.608E-03	4.916E-04	1.000E-03	3.587E-04	4.608E-03	2.505E-04	0.783	0.082	2127.263
**BY2008**	**FFL**	**animal**	**4.090E-03**	**5.067E-04**	**1.000E-03**	**3.864E-04**	**5.090E-03**	**2.639E-04**	**0.804**	**0.078**	**1978.238**
BY2008	Site, FFL	animal	3.469E-03	5.018E-04	1.000E-03	3.586E-04	4.469E-03	2.619E-04	0.776	0.085	2020.511
BY2009 NPTH-NP	None	animal	1.055E-03	7.617E-04	1.240E-03	6.816E-04	2.295E-03	3.535E-04	0.460	0.307	227.775
**BY2009 NPTH-NP**	**FFL**	**animal**	**1.000E-03**	**1.075E-03**	**1.139E-03**	**7.807E-04**	**2.139E-03**	**4.967E-04**	**0.468**	**0.428**	**146.904**
BY2009 NPTH-SP	None	animal	2.014E-03	1.223E-03	2.185E-03	1.046E-03	4.196E-03	6.660E-04	0.479	0.260	194.519
**BY2009 NPTH-SP**	**FFL**	**animal**	**4.363E-03**	**3.008E-03**	**1.081E-03**	**1.865E-03**	**5.444E-03**	**1.418E-03**	**0.801**	**0.379**	**134.413**
BY2009 LG	None	animal	1.641E-03	4.784E-04	1.028E-03	3.393E-04	2.669E-03	2.644E-03	0.615	0.141	673.702
**BY2009 LG**	**FFL**	**animal**	**1.732E-03**	**6.317E-04**	**1.000E-03**	**4.035E-04**	**2.732E-03**	**3.326E-04**	**0.634**	**0.173**	**582.962**
BY2009 CF	None	animal	1.834E-03	1.278E-03	1.820E-03	1.093E-03	3.654E-03	5.796E-04	0.502	0.316	205.072
**BY2009 CF**	**FFL**	**animal**	**3.184E-03**	**2.061E-03**	**1.000E-03**	**1.351E-03**	**4.184E-03**	**9.821E-04**	**0.761**	**0.357**	**137.144**
BY2009 NLV-E	None	animal	2.100E-03	7.028E-04	1.000E-03	5.803E-04	3.100E-03	3.551E-04	0.677	0.193	425.270
**BY2009 NLV-E**	**FFL**	**animal**	**2.018E-03**	**7.330E-04**	**1.000E-03**	**5.821E-04**	**3.018E-03**	**3.748E-04**	**0.669**	**0.202**	**379.640**
BY2009 NLV-W	None	animal	1.013E-02	3.922E-03	1.000E-03	2.841E-03	1.113E-02	2.122E-03	0.910	0.260	404.872
**BY2009 NLV-W**	**FFL**	**animal**	**1.723E-03**	**6.841E-04**	**1.000E-03**	**4.963E-04**	**2.723E-03**	**3.579E-04**	**0.633**	**0.200**	**398.816**
BY2009	None	animal	2.725E-03	3.631E-03	1.000E-03	2.763E-04	3.725E-03	1.935E-04	0.732	0.078	2134.503
BY2009	Site	animal	2.350E-03	3.325E-04	1.000E-03	2.482E-04	3.350E-03	1.760E-04	0.701	0.079	2138.303
**BY2009**	**FFL**	**animal**	**2.572E-03**	**4.020E-04**	**1.000E-03**	**2.882E-04**	**3.572E-03**	**2.050E-04**	**0.720**	**0.087**	**2002.464**
BY2009	Site, FFL	animal	2.910E-03	4.820E-04	1.000E-03	3.341E-04	3.910E-03	2.444E-04	0.744	0.093	1986.712
All BYs	none	animal	3.644E-03	2.758E-04	1.000E-03	2.017E-04	4.644E-03	1.476E-04	0.785	0.045	6299.395
All BYs	BY	animal	3.559E-03	2.724E-04	1.000E-03	1.981E-04	4.590E-03	1.456E-04	0.781	0.046	6305.835
All BYs	Site	animal	3.321E-03	2.663E-04	1.000E-03	1.905E-04	4.321E-03	1.411E-04	0.769	0.047	6363.315
**All BYs**	**FFL**	**animal**	**3.439E-03**	**2.742E-04**	**1.000E-03**	**2.742E-04**	**4.439E-03**	**1.448E-04**	**0.775**	**0.047**	**6151.370**
All BYs	BY, Site, FFL	animal	3.119E-03	2.705E-04	1.000E-03	1.882E-04	4.119E-03	1.406E-04	0.757	0.049	6217.741
